# Cerebrovascular Thrombosis in Pediatric Ulcerative Colitis: A Case Report

**DOI:** 10.3390/reports8010022

**Published:** 2025-02-14

**Authors:** Naire Sansotta, Fabiana Di Stasio, Angela Amoroso, Lorenzo D’Antiga

**Affiliations:** 1 Pediatric Hepatology, Gastroenterology and Transplantation, Papa Giovanni XXIII Hospital, 24127 Bergamo, Italy; a.amoroso@asst-pg23.it (A.A.);; 2 Department of Pediatrics, University of Milano-Bicocca, 20126 Milan, Italy; 3 Department of Medicine and Surgery, University of Milano-Bicocca, 20126 Milan, Italy

**Keywords:** thrombosis, children, inflammatory bowel disease

## Abstract

**Background and Clinical Significance**: Venous thromboembolism (VTE) is a severe extra-intestinal manifestation that can complicate the course of inflammatory bowel disease (IBD). Among pediatric patients, cerebral thrombosis (CT) is the most common form of VTE associated with IBD. Magnetic resonance imaging (MRI) remains the gold standard for diagnosing cerebral venous thrombosis, allowing visualization of flow absence and intraluminal thrombus. Prompt initiation of treatment with low-molecular-weight heparin (LMWH) is crucial to prevent complications. Follow-up imaging is essential to evaluate venous recanalization and guide therapy duration. However, data on cerebral thrombosis in pediatric patient with IBD remain scarce. **Case Presentation**: We report the case of a 12-year-old boy with a known history of ulcerative colitis who presented to the emergency room (ER) with a two-day history of headache and vomiting. One month prior to the ER visit, he experienced an IBD flare confirmed through clinical, biochemical, and endoscopic evaluation and was subsequently started on oral corticosteroids. Neurological examination was unremarkable; however, given the persistence of severe headache, a brain MRI was performed, leading to a diagnosis of cerebral venous thrombosis. Anticoagulation therapy with LMWH was initiated immediately. Follow-up imaging with contrast-enhanced MR venography four months later revealed partial resolution of the thrombosis. The patient continued long-term anticoagulation therapy for a total duration of 12 months. **Conclusions**: Cerebral venous thrombosis is a serious complication of IBD, particularly in pediatric patients. Clinicians should consider this diagnosis in any child with IBD presenting with persistent headache, even in the absence of focal neurological signs. Early diagnosis and prompt anticoagulation therapy are key to improving outcomes in these patients.

## 1. Introduction and Clinical Significance

Venous thromboembolism (VTE) is a severe extra-intestinal manifestation that can complicate the course of inflammatory bowel disease (IBD). Its prevalence ranges between 0.5% and 6% in the IBD population [1]. Patients with IBD have a threefold increased risk of thromboembolic complications compared to the general population, with the absolute risk of VTE being lower in children than in adults (9 events per 10,000 patient-years in children vs. 24 in adults) [2]. The pathogenesis of thrombosis in IBD is multifactorial, with several mechanisms driven by active inflammation contributing to a shift toward a prothrombotic state. The role of bowel and systemic inflammatory burden in the development of VTE is further supported by studies demonstrating a higher incidence of VTE during the first year following an IBD diagnosis [3].

Cerebral venous thrombosis (CVT) is the most common form of VTE in pediatric patients with IBD, accounting for 54% of cases, followed by thrombosis of limb (26%) and abdominal (20%) vessels. Notably, the lateral sinus and superior sagittal sinus are the most frequently affected venous sites in cerebral thrombosis [4]. Neurological symptoms associated with cerebral thrombosis can vary widely, with a sudden onset of global headache being the most common presentation [5]. In patients with clinical suspicion of CT, urgent neuroimaging with contrast-enhanced magnetic resonance imaging (MRI) is essential as the initial diagnostic step. MRI is the gold standard for diagnosing cerebral venous thrombosis, allowing for the identification of absent flow and the presence of intraluminal thrombus. However, these findings may not always be readily apparent [6]. Contrast-enhanced MR imaging can improve the visualization of cerebral venous channels, increasing diagnostic accuracy.

The indication for a comprehensive thrombophilia workup in pediatric patients remains a subject of debate [7]. Management of cerebral venous thrombosis involves prompt initiation of anticoagulation therapy with low-molecular-weight heparin (LMWH), followed by ongoing monitoring to determine the duration of therapy. Personalized risk stratification is crucial for assessing the risk of recurrent thrombotic events and determining the need for secondary prophylaxis [2].

## 2. Case Presentation

A 12-year-old boy with a known history of ulcerative colitis presented to the emergency room (ER) with a two-day history of headache and vomiting. His past medical and family history was unremarkable. No extra-intestinal manifestations were reported. His initial diagnosis of ulcerative colitis was established one year earlier based on frequent abdominal pain, bloody diarrhea, and colonoscopy findings consistent with left-sided colonic inflammation (Paris classification E2). Thus, he was started on oral mesalazine (75 mg/kg/daily), which provided symptomatic relief.

One month prior to the ER visit, biochemical tests revealed persistent iron deficiency anemia (hemoglobin 6.3 g/dL, mean corpuscular volume 55.7 fL, serum ferritin 2 ng/mL) and elevated fecal calprotectin levels (888 mcg/g). PUCAI was 35, consistent with moderate disease. Abdominal ultrasound showed a mild thickening of the left colon (5 mm), and endoscopic re-evaluation revealed severe inflammation throughout the entire colon. Consequently, oral corticosteroid therapy was initiated.

Upon presentation to the ER, physical examination was unremarkable, with normal vital signs. Biochemical tests showed thrombocytosis, mild anemia, elevated inflammation markers, and D-dimer was slightly elevated. A fundus oculi examination was negative. A mild depressive state was noted during the evaluation. However, due to the persistence of symptoms despite analgesic therapy, the patient was admitted to our hospital for further assessment.

**Figure 1:**
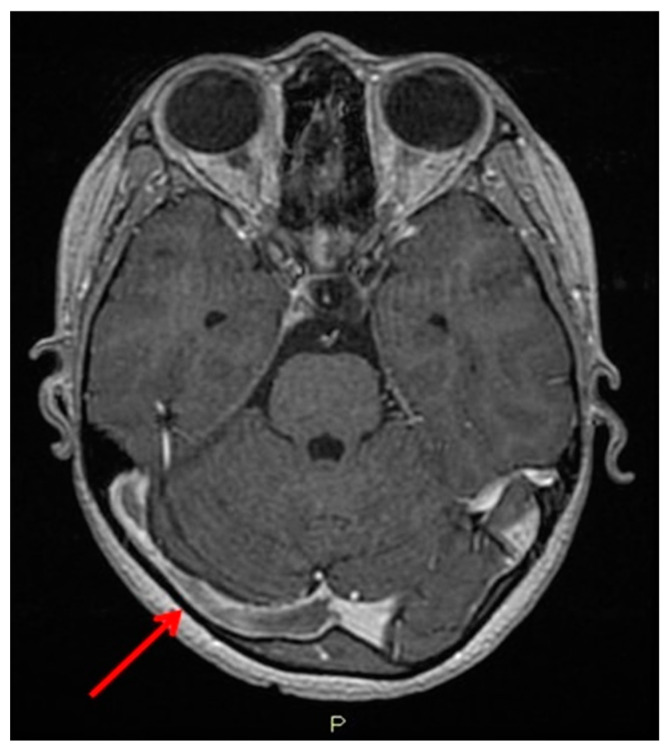
Contrast-enhanced MR venography: the red arrow shows an extensive sinus thrombosis involving the right transverse sinus.

Magnetic resonance angiography (MRA) of the intracranial system revealed an old thrombosis of the superior sagittal sinus. Subsequent contrast-enhanced MR venography confirmed a recent thrombosis involving the right transverse sinus, sigmoid sinus, and superior sagittal sinus, along with evidence of the previously noted superior sagittal sinus thrombosis (Figure 1).

**Figure 2:**
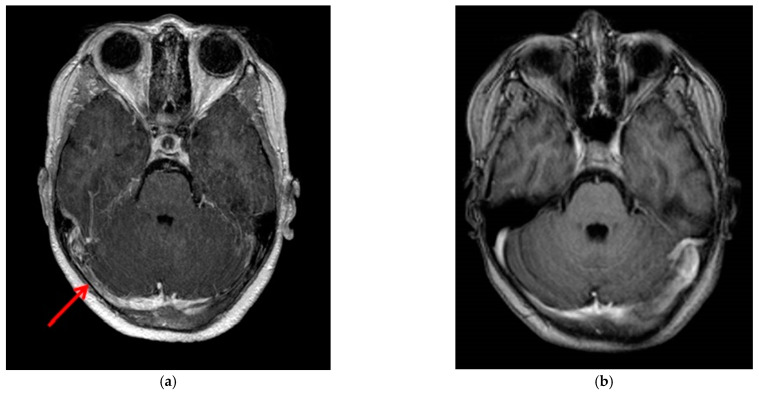
(**a**) Follow-up contrast-enhanced MR venography at month 4. The red arrow shows partial resolution of thrombosis in the right transverse sinus. (**b**) Follow-up contrast-enhanced MR venography at month 12 demonstrates complete resolution of the thrombosis.

Anticoagulation therapy with low-molecular-weight heparin (LMWH) was initiated promptly. A comprehensive thrombophilia workup, including tests for anticardiolipin antibodies, homocysteine levels, protein C, protein S, anti-beta2 glycoprotein-I antibodies, and genetic testing for thrombophilia mutations (polymorphic variants G1691 A of factor V, G20210A of prothrombin, and C677T of methylentetrahydrofolate reductase), yielded negative results. Four months after the initial episode, follow-up contrast-enhanced MR venography showed partial resolution of the thrombosis (Figure 2a). The patient continued long-term anticoagulation therapy for a total of 12 months.

During the 12-month follow-up, repeat MR venography demonstrated complete resolution of the thrombosis (Figure 2b), and anticoagulation therapy was subsequently discontinued.

## 3. Discussion

Cerebral vascular thrombosis (CVT) represents the most common form of VTE in children with IBD with a significant risk of morbidity. The pathogenesis of CVT in patients with IBD is multifactorial, with several mechanisms triggered by active inflammation contributing to a prothrombotic state [8]. Active disease is the primary risk factor for VTE, with acute flares present in 82% of reported cases in patients with IBD [9,10]. Among patients with IBD, corticosteroid use has been associated with an increased risk of VTE, whereas mesalamine and anti-TNF therapy do not appear to increase this risk [11].

The neurological presentation of CVT can be highly variable, with sudden-onset symptoms being common. Persistent, predominantly global headache is the most frequent symptom, occurring in approximately 90% of cases. Other manifestations include vomiting, papilledema, focal neurological deficits, and seizures [5].

Among biochemical blood tests, D-dimer seems to work well in an emergency room setting in excluding VTE. Elevated D-dimer has been reported to have high diagnostic specificity and sensitivity, but recent studies have questioned D-dimer’s value in excluding VTE and its value in follow-up [12].

Neuroimaging (CT and MRI) is fundamental in the diagnosis of CVT. Of note, CT in the acute stage seems to display higher sensitivity while the MRI displays higher specificity [13]. In addition, MRI offers superior delineation of parenchymal abnormalities compared to computed tomography (CT) and avoids the radiation exposure associated with CT imaging [6,14]. As a problem-solving modality, CT remains the first-line imaging method in the emergency department setting, especially in patients with unspecific neurologic symptoms or where MRI is contraindicated or not available. Conversely, MRI should be the best choice for the diagnosis (when available) and the follow-up cases of diagnosed thrombosis [13].

The indications for a thrombophilia workup in pediatric patients with CVT remain controversial. It is generally recommended if the results would influence the treatment approach. Genetic and environmental factors interact in determining the risk of VTE. In a recent large, pooled analysis, no effect was found for C677T methylentetetrahydrofolate reductase on VTE, while G1691A of factor V and G20210 of prothrombin were confirmed to be moderate risk factors [8]. Some authors suggest thrombophilia screening in cases of the first thrombosis episode, recurrent thrombosis, or a family history of VTE before the age of 50 [15,16].

Antithrombotic therapy is essential to achieve venous recanalization, prevent thrombus propagation, and address the underlying prothrombotic state to reduce the risk of recurrence. A meta-analysis demonstrated that therapeutic anticoagulation with heparin is safe in patients with active colitis, without a significant increase in bleeding complications [17]. For the acute management of VTE, low-molecular-weight heparin (LMWH) remains the most commonly used therapy. The recommended dose is 100 IU/kg administered twice daily, and treatment should begin as soon as the diagnosis is confirmed. Warfarin has traditionally been used for subacute and chronic management due to its oral administration and long half-life [18].

Follow-up imaging to assess venous recanalization is necessary to determine the appropriate duration of anticoagulation therapy. Pediatric data on this topic are limited, but current guidelines suggest continuing anticoagulation for a minimum of three months and not exceeding 12 months [2,7,19]. Recurrent thrombosis occurs in up to 25% of cases, with 70% of these recurrences involving the same site as the initial episode. Secondary prophylaxis should be considered in pediatric patients with IBD with a history of thrombosis, particularly during moderate-to-severe IBD flares or hospitalization for major surgery [20]. There is ongoing debate about the need for primary prophylaxis in children with IBD. The European Society for Pediatric Gastroenterology, Hepatology, and Nutrition (ESPGHAN) recommends primary prophylaxis with LMWH (100 IU/kg/day) in adolescents with one or more risk factors (e.g., smoking, oral contraceptive use, complete immobilization, central venous catheter, obesity, concurrent significant infection, known prothrombotic disorder, previous VTE, or family history of VTE) and in prepubertal children with at least two risk factors [2].

There is little literature on the long-term outcome and implications for quality of life. Mortality trend was low with earlier diagnosis and aggressive treatment [21]. A small case series report showed that most of them had normal cognitive and physical development, although mild cognitive deficits or decreased physical and psychosocial well-being can occur [22].

## 4. Conclusions

Cerebral sinus and vein thrombosis is a rare but serious complication of IBD. It should be considered in any patient with IBD presenting with persistent severe headache and focal or diffuse neurological symptoms, particularly in the context of a recent IBD diagnosis, acute flare, or use of prothrombotic medications such as corticosteroids.

In patients with clinical suspicion of cerebral thrombosis, urgent neuroimaging with MRI (if available) is essential as the initial diagnostic step. Management involves prompt initiation of anticoagulation therapy with low-molecular-weight heparin (LMWH), regular follow-up to determine the appropriate duration of therapy, personalized risk stratification to prevent thrombotic events, and consideration of secondary prophylaxis in high-risk patients.

## Data Availability

The original contributions presented in this study are included in the article. Further inquiries can be directed at the corresponding author.

## Abbreviations

Venous thromboembolism (VTE); inflammatory bowel disease (IBD); mean cell volume (MCV); magnetic resonance angiograph (MRA); low-molecular-weight heparin (LMWH); methylene tetrahydrofolate reductase (MTHFR); factor V Leiden (FVL); cerebral vein thrombosis (CVT); European Society for Pediatric Gastroenterology Hepatology and Nutrition (ESPGHAN).
